# Investigation on Centrifugally Spun Fibrous PCL/3-Methyl Mannoside Mats for Wound Healing Application

**DOI:** 10.3390/polym15051293

**Published:** 2023-03-03

**Authors:** Soloman Agnes Mary, Naisini Ariram, Arun Gopinath, Senthil Kumar Chinnaiyan, Iruthayapandi Selestin Raja, Bindia Sahu, Venkateshwarapuram Rengaswami Giri Dev, Dong-Wook Han, Balaraman Madhan

**Affiliations:** 1 Centre for Academic and Research Excellence, CSIR-Central Leather Research Institute Adyar, Chennai 600020, India; 2 BIO-IT Foundry Technology Institute, Pusan National University, Busan 46241, Republic of Korea; 3 Department of Textile Technology, Anna University, Chennai 600025, India; 4 Department of Cogno-Mechatronics Engineering, College of Nanoscience & Nanotechnology, Pusan National University, Busan 46241, Republic of Korea

**Keywords:** centrifugal spinning, ultrafine porous fibers, cassia auriculata, polycaprolactone, wound healing

## Abstract

Fibrous structures, in general, have splendid advantages in different forms of micro- and nanomembranes in various fields, including tissue engineering, filtration, clothing, energy storage, etc. In the present work, we develop a fibrous mat by blending the bioactive extract of Cassia auriculata (CA) with polycaprolactone (PCL) using the centrifugal spinning (c-spinning) technique for tissue-engineered implantable material and wound dressing applications. The fibrous mats were developed at a centrifugal speed of 3500 rpm. The PCL concentration for centrifugal spinning with CA extract was optimized at 15% *w*/*v* of PCL to achieve better fiber formation. Increasing the extract concentration by more than 2% resulted in crimping of fibers with irregular morphology. The development of fibrous mats using a dual solvent combination resulted in fine pores on the fiber structure. Scanning electron microscope (SEM) images showed that the surface morphology of the fibers in the produced fiber mats (PCL and PCL-CA) was highly porous. Gas chromatography–mass spectrometry (GC-MS) analysis revealed that the CA extract contained 3-methyl mannoside as the predominant component. The in vitro cell line studies using NIH3T3 fibroblasts demonstrated that the CA-PCL nanofiber mat was highly biocompatible, supporting cell proliferation. Hence, we conclude that the c-spun, CA-incorporating nanofiber mat can be employed as a tissue-engineered construct for wound healing applications.

## 1. Introduction

Over the last few decades, tissue engineering scaffolds with different bioactive agents for wound healing have gained much attention among the research fraternity. Tissue engineering scaffolds have been used for various applications such as reconstructing traumatic injuries, healing acute, chronic, and surgical site wounds that are prone to infection, as dermal substitutes, or as dressing materials [1]. Healing is a natural process in our body to reconstruct the dermal or epidermal tissue damaged due to injury. Depending on the severity of the wound and the underlying diseases, the wound healing cascade may differ from one individual to another [2]. In particular, healing may be delayed in patients with diabetes or other related diseases, and such long-standing wounds are commonly called chronic wounds. Diabetic wounds are prone to delayed healing due to the impairment of intrinsic factors such as vascular problems or other complicated side effects such as diabetes and extrinsic factors such as microbial infection or pressures created at the wound site. Chronic wounds fail to progress through the normal healing cascade due to infections or age-related factors that affect the signaling pathway of the healing process [3]. Extensive research was done and is also emerging in biomaterials that aim to reduce healing time in chronic, infected, and traumatic wounds.

An ideal wound dressing or polymeric-based fibrous dressing should be highly porous, non-toxic, and biodegradable, and also should possess better surface compatibility, interconnected network to mimic ECM, mechanical strength, adequate permeability to offer moisture balance, gas exchange, and nutritional exchange. Furthermore, it should prevent pathogenic microbial flora at the wound site. Moreover, it should rapidly promote the wound healing process at the wound site [4]. Nanofibrous wound dressings, collagen dressings, occlusive dressings, colloidal dressings, foams, hydrogels, and bioactive dressings are available in the market for different applications [5]. Nanofibrous dressings mimic our tissue’s extracellular matrixes (ECM), which possess the ideal characteristics of a wound dressing material and are superior in surface area to volume ratio and porosity that support tissue regeneration [6]. The nanofibrous membranes are produced by different methods, such as phase separation, melt blowing, drawing, electrospinning, and centrifugal spinning. Fibers ranging from a few micrometers to nanometer levels can be fabricated for various applications in different fields [7]. The centrifugal spinning technique currently gains attention and interest in nanofibers and ultrafine fibers production. Ultrafine fibers were developed using melt blowing and flash spinning techniques since 1950s. Fibers with diameters less than 3 μm are referred to as ultrafine fibers. A fiber less than 0.1 denier d is referred to as super ultrafine fiber or a short version of microfiber [8]. It is a reliable and cost-effective technology that maximizes production and minimizes time consumption.

The fine fibrous materials were produced using the centrifugal spinning technique using different synthetic polymers viz., polycaprolactone (PCL), polylactic acid (PLA), polyvinyl pyrrolidone (PVP), and other synthetic polymers [9,10,11]. In a centrifugal spinning setup, when the polymeric solution is fed into the solvent reservoir by applying centrifugal force, the spinneret ejects the polymeric solution at the critical value of the rotational speed. The polymeric solution exceeds the surface tension, extends to form fine to ultrafine fibers, and gets collected on the collector circumference [12,13,14]. Elongation of the polymeric solution and solvent evaporation causes the liquid jets to dry and form a continuous fibrous morphology. The fiber formation depends on solvent evaporation, the viscoelasticity of the solution, and the applied centrifugal force. The spinneret can generate fibers from the macro to nano range depending on the ability of the polymeric solution to form fibers [15,16,17,18]. Various researchers have employed the incorporation of bioactive compounds to develop bioactive scaffolds. Incorporating herbal extracts as bioactive compounds in biomaterials using electrospinning and other nanofiber production techniques to develop a bioactive herbal dressing has been mentioned in various studies [19,20].

India has been a significant research consortium hub for herbal medicine practice and facilitating herbal research-related works to enhance the usage of herbal drugs. Polymeric biomaterials have many applications as therapeutics or bioactive vehicles in medicinal scaffolds, implants, and tissue-engineered implants to treat damaged tissues or organs. Synthetic or natural polymeric plant extract-based scaffolds have shown a significant range of biocompatibility and regeneration ability in the target site without harm to the surrounding tissue or organs [21]. This study develops a biomaterial using *Cassia auriculata* (CA) extract incorporation. The CA is a shrub commonly known as “avaram” in Tamil, with attractive yellow flowers used for treatments in skin disorders, diabetes, rheumatism, and conjunctivitis. CA is a medicinal plant used from ancient times as a therapeutic agent for various ailments, including stomach aches and skin disease; its fruits and leaves are used as an anti-diabetic medicine and possess other medicinal values [22,23]. Various studies have reported that CA contains important phytochemicals that can biologically stimulate antibacterial, antioxidant, anti-inflammatory, wound healing, anti-diabetic, and anti-malarial activities. Pharmacological studies on different parts of the plant extracts, such as leaves, flowers, roots, and whole plant extract, can cure and act against various infectious diseases and microbial pathogens, mainly to heal diabetic wounds [23,24,25]. Although screening of phytochemicals and their topical applications for acute and diabetic wound healing have been well-studied, extract-containing fibrous biomaterials need to be explored. In the present work, CA was extracted using chloroform and methanol as solvent, and the GC-MS analysis reported the presence of an abundant phytochemical derivative 3-methyl mannoside in the extract, which may be an essential compound with respect to its antimicrobial properties [26].

The study also explores the possibility of spinning PCL in chloroform: methanol as a dual solvent combination using C-spinning techniques. C-spinning technology is the simplest technology for achieving higher fiber production in less time. SiO_2_ fibers developed using centrifugal spinning techniques for water adsorption property were studied by Ludek et al. [27]. Centrifugally spun matrices incorporating natural polymer chitosan/silver as drug and PCL/PVP/tetracycline were loaded to study the efficiency of c-spun fibers as a drug delivery vehicle [13,15]. The present work deviates from the previous studies by elucidating the feasibility of developing a herbal extract incorporating mats using c-spinning using dual solvent technique with highly porous fiber morphology for tissue engineering and wound healing applications. Optimization of PCL with CA extract in the dual solvent fiber production was carried out and studied for its spinnability and fiber formation. The study also elucidates the physical and biological characteristics of the developed material for tissue engineering and wound healing applications.

An attempt has been made to develop a PCL polymeric dressing material incorporated with CA extract (PCL-CA) using a centrifugal spinning technique to create a wound healing material. The PCL is a synthetic biodegradable semi-crystalline FDA-approved polymer, which can be easily spun into fibers and is one of the well-explored polymers in biomaterials [28,29]. Additionally, electrospun PCL fibers are suitable for treating acute and chronic wounds [6]. The present research focuses on the feasibility of spinning CA extract with PCL using centrifugal spinning and characterizing the developed fibers. The study also explores the possibility of spinning PCL in chloroform: methanol as dual solvent combination using C-spinning techniques. The study also elucidates the physical and biological characteristics of the developed material for tissue engineering and wound healing applications.

## 2. Materials and Methods

### 2.1. Materials

Polycaprolactone (PCL) (Mw: 80,000) was procured from Sigma Aldrich (Chennai, India). CA powder was procured from a locally available herbal medicine shop (Bala Exports & Imports, Chennai, India). Chloroform and methanol of medical grade were purchased from Sisco Research Laboratories Pvt. Ltd. (Mumbai, India), and used as received.

### 2.2. Preparation of CA Extract

The CA extract was prepared using the solvents chloroform and methanol in a ratio of 1:1. Twenty-five grams of herbal (CA) powder was taken and refluxed in chloroform: methanol (200 mL) mixture in a Soxhlet apparatus for 35 cycles. Then, the crude extract was collected, concentrated, and stored for further experiments.

### 2.3. Preparation of Solution for Centrifugal Spinning

PCL was dissolved in chloroform: methanol as solvent was used for centrifugal spinning at various concentrations and was optimized in the centrifugal spinning machine with and without heating the polymeric solution before spinning. PCL (*w*/*v*) solutions were prepared at different concentrations, such as 10%, 12%, and 15%. For the fiber production in C-spinning 10% and 12% PCL solution produced fibers with bead formation and incomplete drying of solvents was observed. Therefore, the PCL concentration was increased further to 15% PCL, and it was found that the solution was highly suitable for producing fibers without beads. Thus, 15% PCL solution was found to be optimal for the production of fibrous mat, and was therefore regarded as optimal for further experiments based on fiber formation. To the optimal PCL concentration, herbal extract was added in various ratios of 0.5%, 1%, 1.5%, and 2%.

### 2.4. Centrifugal Spinning Process

**Figure 1:**
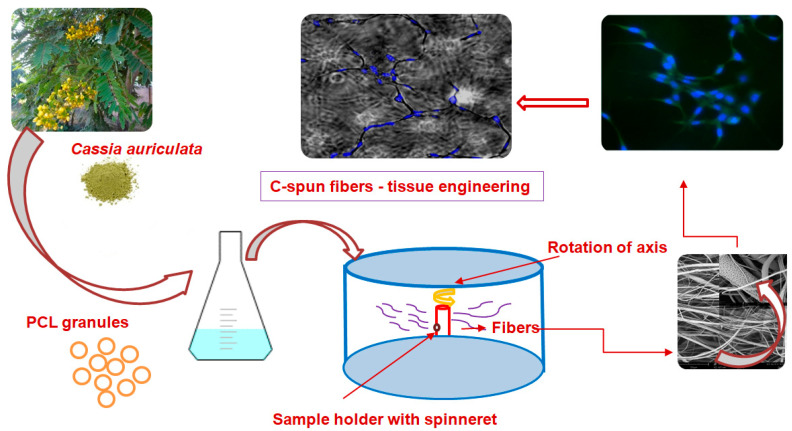
Complete flowchart of the production of fibers through centrifugal spinning.

Centrifugal spinning was carried out in a recently developed centrifugal spinning machine, Umex India Pvt. Ltd. (Chennai, India). In brief, 6 mL of the PCL-CA solution was taken in the sample holder with an orifice of less than 0.1 mm. The solution was placed on the centrifugal spinning machine and spun. The collector was maintained 15–35 cm away from the center of the spinneret. The solution was set to flow at a constant flow rate. When the rpm was increased to 3500, the solution ejecting out through the orifice formed meshes of fluffy fibers ranging from nanometers to tens of micrometers. The fibers formed were stored for further characterization. The complete flowchart of the production of fibers through centrifugal spinning is depicted in Figure 1.

### 2.5. Instrumental Characterization

The morphology of the developed centrifugally spun (c-spun) PCL and PCL-CA fibers were studied by SEM (Phenom ProX Desktop SEM, Thermo Fisher Scientific, Chennai, India) after sputter coating them with gold. The diffraction patterns of the as-prepared fiber mats were analyzed using X-Ray Diffractometer (Rigaku Corporation, Tokyo, Japan). Fourier transform infrared spectroscopy (FTIR)-(FT/IR-4700 Spectrometer, JASCO Inc., Easton, MD, USA) was carried out for the developed PCL- and PCL-CA-incorporating fibers for functional group analysis. The tensile strength of the fibrous mats (INSTRON 3369/J 7257, Bangalore, India) was studied to determine the tensile properties of the developed fibrous materials, as per standard procedure ASTM D887. The thickness of the fibrous materials was measured using a thickness gauge.

Thermogravimetric analysis of the samples was carried out to determine the thermal stability of the developed fibers before and after the incorporation of the herbal extract. TGA was performed using an instrument by TA Instruments, Waters, Austria.

The contact angle of fiber mats was studied using a contact angle meter (Holmarc Opto-Mechatronics Ltd. Kochi, India). A drop of sterile water of about 10 µL was dropped onto the fibrous mat after fixing it with double-sided tape using a micro syringe using the sessile drop method. The image was captured after dropping the water droplet and analyzed for the contact angle with the samples. The experiments were done in triplicates to get concurrent results.

GC-MS analysis was performed to study the presence of phytochemical components in the chloroform-methanolic extract of CA. The experiment was carried out in a GC-MS, Bruker, SCION 8900 TQ system (India Sales Manager, New Delhi, India) comprising GC-MS equipment in the following conditions: Mobile phase: Helium as a carrier gas with a flow rate of 1 mL/min; Column name: DB 5Ms, 30 m × 250 µm × 0.25 µm (calibrated). In the gas chromatography part, the temperature (oven temperature) was raised from 5 °C to 280 °C, and the injection volume was 1 µL. Samples dissolved in chloroform–methanol were run entirely at 50 to m/z mass units. The results were compared and interpreted using the National Institute Standard and Technology (NIST) database.

### 2.6. Protein Denaturation Assay

The protein denaturation assay for the developed mats was performed as described by Gambhire et al., with slight modification [30]. The mats were incubated for 24 h in phosphate-buffered saline (5 mL each), and 2 mL of released extract from each concentration was taken for the study. The released extract was studied for its denaturing efficiency with BSA (bovine serum albumin) protein. The reaction mixture consisted of 0.2 mL of 1% BSA, 4.78 mL of PBS, and 0.02 mL of extract, and PBS solution was used as a control. The reaction mixture was then incubated in a water bath at 37 °C for 15 min and heated at 70 °C for 5 min. The mixture was cooled, and the turbidity was measured using a UV spectrophotometer at 600 nm. Denaturation of protein to inhibition was calculated using the following formula:

(1) $$\mathrm{Percentage}\;\mathrm{Inhibition}\;\left(\%\right)=\frac{\left(\mathrm{Abs}\;\mathrm{Control}-\mathrm{Abs}\;\mathrm{Sample}\right)}{\mathrm{Abs}\;\mathrm{Control}}\times 100$$

### 2.7. Cell Viability Using MTT Assay

The MTT assay was performed on the PCL- and PCL-CA-incorporating fibrous mats using fibroblast NIH3T3 cell lines. The cells were seeded onto the mats, placed in sterile well plates, and grown in Dulbecco’s modified Eagle Medium (DMEM) containing 10% fetal bovine serum. The cultures were maintained in a 5% CO_2_ incubator at 37 °C, and the medium was changed every three days. The cells were cultured on the mats for 1, 3, and 5 days and compared with control wells (without mats). In addition to PCL mats, 1.5% of CA extract was also subjected to proliferation assays at 10 µL, 20 µL, and 30 µL. The absorbance was read at 590 nm in a microplate reader (BioTek Instruments, Winooski, VT, USA). The absorbance values obtained are expressed in optical density (OD).

### 2.8. Cell Morphology and Adhesion Evaluation

The cell attachment and viability on the mats were evaluated using 4′,6-diamidino-2-phenylindole (DAPI) and Calcein AM staining techniques. Fibroblast cells were seeded onto the mats and grown under similar conditions as the MTT assay. The cells were stained with DAPI and Calcein AM at days 1, 3, and 5 of culture. Then, the cells were observed using a fluorescence microscope (Leica, Thermo Fisher Scientific, Chennai, India), and images were taken for analysis.

### 2.9. Statistics

Data are expressed as mean ± SD. Statistical significance was determined by one-way ANOVA using Dunnett’s multiple comparison test. Statistical analyses were performed using Graph Pad Prism 5.01 software. Statistical significance was determined by *p*-values (* *p* < 0.05; ** *p* < 0.01).

## 3. Results and Discussion

The present study elucidates the facile centrifugal spinning of plant extract-incorporating fibrous scaffold for tissue engineering applications. The crude extract of CA possesses a high level of polyphenols, flavonoids, tannins, alkaloids, carbohydrates, and steroids. The extracted CA was subjected to biochemical analysis, and the presence of phytoconstituents was confirmed and provided in Table S1. Meena et al. reported that the methanolic extract of CA has profound anti-inflammatory effects on both acute and chronic wounds, and chloroform extract has antipyretic properties and various antibacterial activities [31].

### 3.1. Identification of CA Extract Compounds

The peaks were marked by the retention time of GC-MS chromatogram with chloroform: methanol extract of CA (Figure S1, Supplementary Materials). The compounds present, along with their respective retention times, are listed in Table 1. The qualitative analysis showed the presence of five compounds in chloroform: methanol extract, viz., L-alanyl-L-methionine (with a retention time of 6.702), n-hexylmethylamine (16.641), 3-methyl mannoside (23.755), di-sec-butyl-phthalate (28.881) and N-Methyl-N-octadecylamine (37.086) [32].

The results of GC-MS analysis revealed that 3-methyl mannoside was predominantly present in the CA extract. 3-methyl mannoside or aglycone is a glycoside of a flavonoid of plant origin that possesses versatile biological activities, including antioxidant, anti-inflammatory, anti-diabetic, anti-bacterial, antifungal, antiviral, and other biological effects [33]. Generally, natural flavonoids occur in the form of C-glycosides or O-glycosides. The glycoside found in the extract was O-glycoside, as it is methylated at the first position [34]. It has been reported in several studies that 3-methyl mannoside has the efficacy in inhibiting bacterial growth by lectin conjugate binding pathway and is also used for drug delivery by targeting antigen-presenting cells through mannose receptors [35].

### 3.2. Optimization of PCL Concentration in Fibrous Mats

A number of fibrous mats were produced from PCL polymeric solution at concentrations of 10%, 12%, and 15% (*w*/*v*). The optimization of PCL concentration with a fine appearance was investigated using the formulation cited in the literature [15]. Fiber formation using the c-spinning method was performed with and without mild heating of the PCL solutions before spinning. Among the three different concentrations, 15% (*w*/*v*) PCL with mild heating resulted in good fiber formation without beads. The solution without heating also developed good fiber morphology. However, the slow solvent evaporation rate observed more crimping of fibers. The solution subjected to mild heating resulted in less crimped morphology [15,36], which may be attributed to a higher solvent evaporation rate. The polymer solution was pre-heated externally, as the heating provision was absent in the centrifugal spinning setup. Optical images of PCL- and PCL-CA-incorporating c-spun fibers are provided in Figure S2.

### 3.3. Surface Morphology of Fibrous Mats

**Figure 2:**
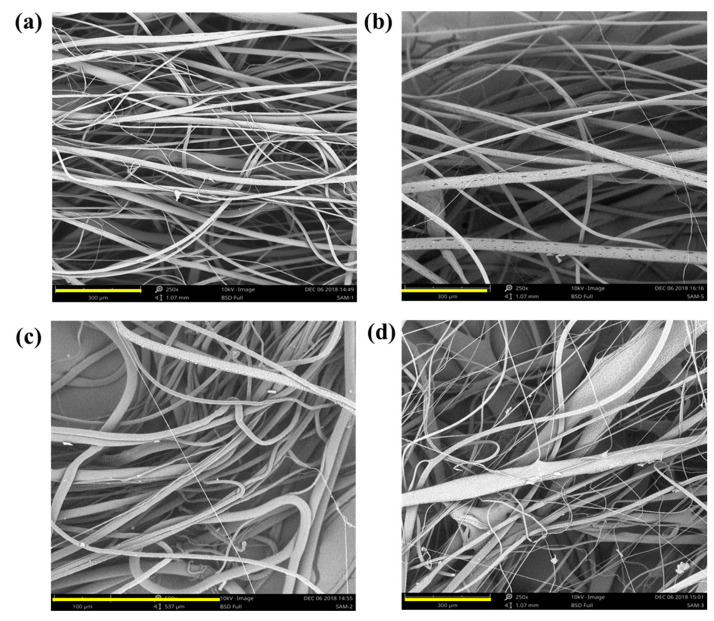
SEM images of c-spun PCL and PCL-CA fibrous mats. (**a**) PCL without heating, (**b**) PCL with heating, (**c**) PCL-CA without heating, and (**d**) PCL-CA with heating. Scale bar is 300 µm.

The morphology of fibers is usually controlled by adjusting the intrinsic properties of polymeric fluids, such as viscosity, molecular weight of the polymer, concentration, rotating speed, centrifugal force applied, additives, nozzle head diameter, and collector distance. The surface morphology of the developed c-spun fibers was observed using SEM, as shown in Figure 2.

The surface morphology of the fiber in the PCL and PCL-CA mat appeared as a highly porous gauze-like fibrous mesh. Upon increasing the concentration of the extract, the fiber morphology was disturbed and became more crimped and sticky in nature and appearance. The surface morphology of the developed c-spun fibers showed a highly porous surface with randomly aligned fibers. The porous fiber morphology and the alignment of fibers on the fiber surface depend on the use of a dual solvent combination, environmental humidity, and also the solvent evaporation rate, which depends inherently on the rotating speed and polymer viscosity upon drying, resulting in a higher diameter of the fiber [10].

The solvent temperature may also lead to a higher solvent evaporation rate, forming more pores and higher fiber formation. The centrifugation speed applied to produce fibers was optimally around 3500 rpm. As the fibers are produced within a specific rpm limit, which increases the fiber diameter, a further increase in centrifugal force resulted in decreased fiber diameter. The micrograph also showed some crimped morphology of fibers, which may be due to insufficient solvent drying. Rayleigh’s solution instabilities may also have resulted in the slight crimping morphology of fibers. Pores can be observed on the entire surface of the fibers due to the polymeric solution’s higher evaporation rate and rapid fiber formation. Even pores on the fiber surface may be attributed to the gradual evaporation of solvent from the surface matrix. The highly porous morphology of any biological materials for tissue engineering applications is very helpful in cell growth by influencing the growth pattern [37,38,39,40,41]. The porous morphology is needed on the wound healing mats for the adherence of the cells or to enable the exchange of nutrients and gases through the mat network, thereby accelerating the wound healing efficacy at the wound site. Thus, the developed porous fibrous morphology of the mat can enhance the wound healing process in tissue engineering applications [42].

**Figure 3:**
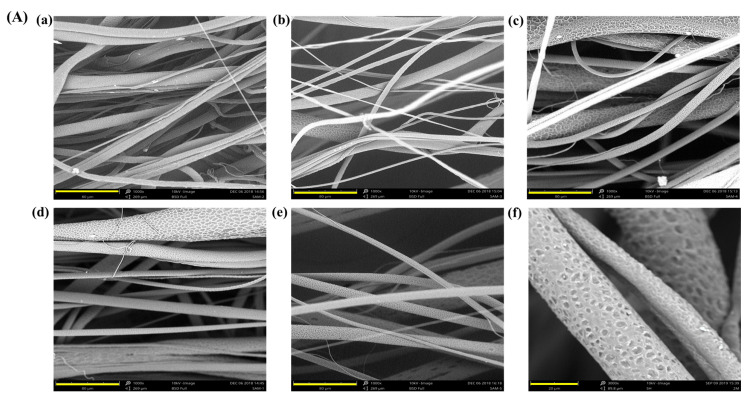

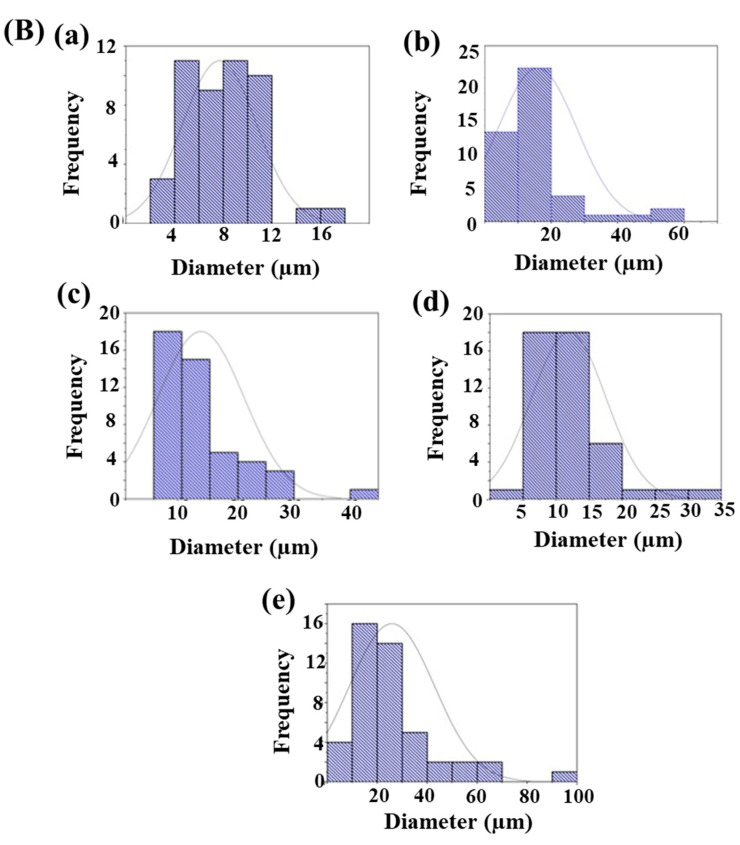
(**A**) SEM observation of c-spun PCL and PCL-CA fibers. (**a**) PCL (Control), (**b**) PCL + 0.5% CA, (**c**) PCL + 1% CA, (**d**) PCL + 1.5% CA, (**e**) PCL + 2% CA and (**f**) an enlarged image of fibers showing porous appearance. Scale bar of (**a**–**e**) is 80 µm and scale bar of (**f**) is 20 µm. (**B**) The diameter distribution plot of c-spun PCL and PCL-CA fibers. (**a**) PCL (Control), (**b**) PCL + 0.5% CA, (**c**) PCL + 1% CA, (**d**) PCL + 1.5% CA, and (**e**) PCL + 2% CA.

Compared to the electrospinning process, the major advantage of the centrifugal spinning process is the high production rate of the polymer fibers with less time consumption, which is very cost-effective. It can produce a large quantity of micro to nanofibers on a bulk scale in a significantly shorter time. Figure 3 shows the SEM micrographs of ultrafine fibers produced from heat-induced PCL melt solution with chloroform and methanol as solvents with and without the incorporation of herbal extract. The average fiber diameter was calculated using Image J software and was found to be 1.7, 1.8, 1.2, 1.2, 1.4, and 1.76 µm. Upon increasing the concentration of the extract in the polymer solution, the PCL-CA matrices exhibited aggregation of fibers compared to pure PCL fibers. The PCL-CA fiber morphology showed an aligned fiber structure, the same as plain PCL with a fine porous structure [43]. Images of the PCL- and CA-incorporating c-spun fibers with various concentrations of CA, viz., 0.5% CA, 1% CA, 1.5% CA, and 2% CA, along with fiber diameter, are given in Figure 3A.

The porosity of the developed mats was determined using the method cited in the literature [44], and their results have been provided in Table 2.

Uniform evaporation of solvents resulted in the development of fine pores on the fiber structure. It was observed that mild heating of PCL and PCL-CA polymeric solutions had aided the fiber formation with well-defined porous morphology due to the solvents’ high evaporation rate and solvent composition (chloroform and methanol) [45].

### 3.4. Functional Groups and Crystallinity of PCL-CA Fibrous Mats

**Figure 4:**
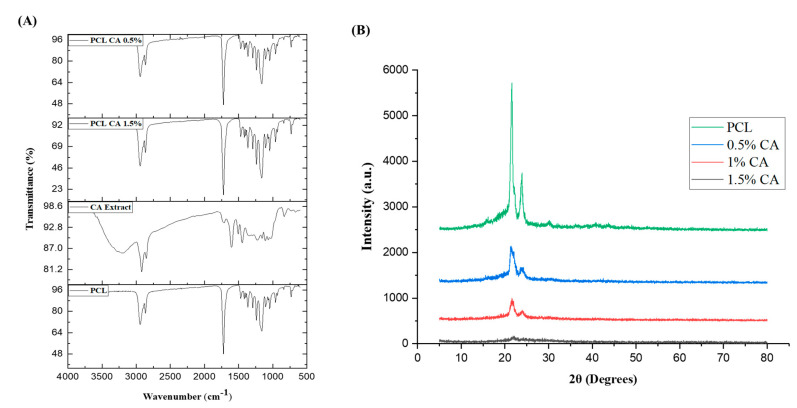
(**A**) FTIR spectra of PCL, CA extract, PCL+ 0.5% CA, and PCL+ 1.5% CA. (**B**) XRD spectra of c-spun PCL and PCL−CA fibrous mats (CA: 0.5, 1, and 1.5%).

The FTIR spectra of the PCL- and CA-incorporating c-spun matrices were studied to find the functional groups of the active compounds present in the extract (Figure 4A). The PCL c-spun matrices exhibited characteristic symmetric and asymmetric peaks at 2830 and 2950 cm^−1^.

The stretching vibrations for ester bond C=O appeared at 1729 cm^−1^, and the CH_2_ bending vibrations occurred at 1350 and 1463 cm^−1^ [46]. The –C-O-O vibration gives characteristic peaks around 1290, 1235, and 1179 cm^−1^. The peaks of symmetric and asymmetric stretching vibrations were observed for C-O-C vibrations at 1050 and 1009 cm^−1^, respectively. The corresponding peaks of PCL-CA matrices were similar to those of PCL, with a shift in the major peaks due to the incorporation of the CA extract. CA-incorporating PCL matrices showed a shift in the peaks of stretching vibrations at 2800 and 2950 cm^−1^. A major shift in the aromatic bending vibrations occurred in the CA-incorporating PCL mats, observed at 1055 and 1100 cm^−1^ for the biologically active compounds present in the extract [22].

The XRD spectra of PCL and PCL-CA matrices are elucidated in Figure 4B. The XRD pattern for the pure PCL showed diffraction peaks at 2θ = 21.5°, 22.1°, and 23.8°, which were assigned to the planes (110), (111), and (200) of PCL, respectively [47]. On the other hand, CA-incorporating PCL matrices exhibited an amorphous region in their planes, suggesting that the extract affects the crystalline nature of PCL. CA-incorporating PCL matrices exhibited an amorphous region in their planes, suggesting that the increase in extract concentration resulted in a gradual decrease in the intensity of the crystalline nature of the PCL, which may be attributed to the presence of bioactive compounds with an amorphous nature.

### 3.5. Thermal Stability, Wettability and Mechanical Properties of Fibrous Mats

**Figure 5:**
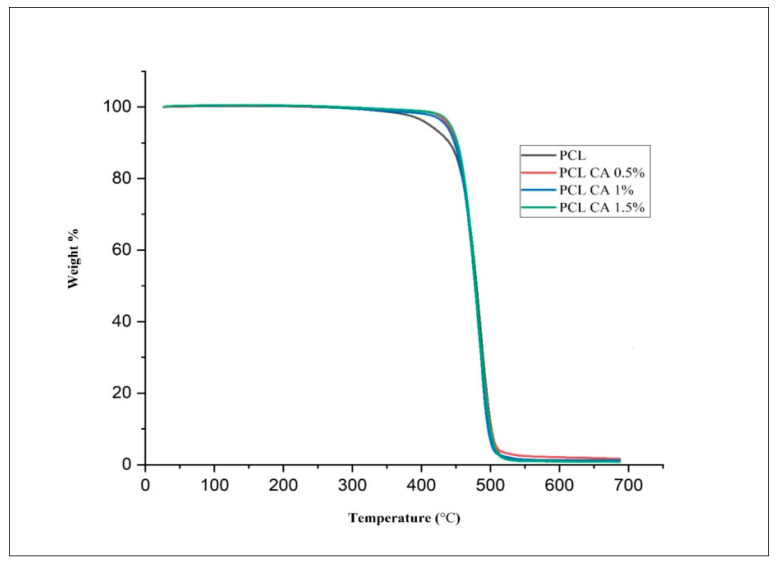
TGA traces of c-spun PCL and PCL-CA fibrous mats (CA: 0.5, 1, and 1.5%).

Thermogravimetric analyses for PCL and PCL-CA matrices were carried out to study thermal stability, as illustrated in Figure 5. The PCL polymer showed initial moisture degradation and weight loss at 369 °C; the second stage of weight loss occurred at 477 °C, and a third stage of weight loss occurred at 556 °C with the elimination of residues. The incorporation of CA extract into the c-spun matrices slightly changed the thermal stability of the matrices, and there is a slight increase in thermal stability compared to pure PCL mats.

The PCL is a biodegradable polymer that is easy to process, with a low melting point, and the Tg falls at approximately 60 °C. It is a polymer that is widely recognized for its suitability for tissue engineering and wound healing applications. The PCL-CA matrices showed two-stage weight loss, where initial weight loss occurred at 420–450 ℃, while the second stage of weight loss matched with the parent polymer. The increase in stability may be attributed to the interaction between amine groups of extract the PCL, as PCL undergoes aminolysis with –NH_2_ groups present in the extract. PCL-CA reflects an increase in stability compared to bare PCL, with PCL exhibiting an initial weight loss at 370 °C and then at 450 °C due to the low volatility of the molecules of the polymer, while the second stage of weight loss resulted in the complete decomposition of the residual polymer.

Wettability is a uniquely important physical property of any wound dressing material. Contact angle analysis, performed using a goniometer, describes a material’s surface energy or the static wettability of any biomaterial. The contact angle value of a material defines the interaction of the material with water or other biological fluids to adhesion, surface interaction, absorption, and permeation. Scaffolds for tissue engineering applications need to possess a certain degree of contact angle or wettability to be biocompatible in order to allow the cell growth, migration, and differentiation of cells on the substrate. The scaffold should not be more hydrophobic, leading to poor adhesion of proteins. A more hydrophilic scaffold can lead to an inability of the cells to form a consistent or smooth monolayer on the surface. The scaffolds for tissue engineering should be moderately wettable or hydrophilic for enhanced biocompatibility and proliferation [48,49]. PCL is a hydrophobic polymer exhibiting contact angle values between 116 and 135°. In this study, pure PCL presented a contact angle of 130°, and the PCL-CA samples with 0.5%, 1%, and 1.5% of CA showed angles of 110°, 100°, and 90°, respectively (Figure S3). The concentration-dependent decrease in contact angle values was observed for PCL-CA due to the incorporation of hydrophilic CA extract.

A tissue-engineered substrate should possess tensile properties comparable to human skin for handling, suturing, and holding dressings on the wound surface. The developed mats have similar tensile properties (2.5–16 MPa) for tissue engineering applications [50]. The mechanical properties of the developed c-spun mats can be understood from the tensile strength, load, elongation at break, and maximum extension values in Table 3. The PCL mats showed a higher tensile strength but lower elongation at break compared to PCL-CA mats. When CA concentration increased from 0.5% to 1.5% in the PCL mat, the tensile strength gradually decreased, and elongation at break increased. Increase in extract concentration resulted in a decrease in tensile strength being observed in the developed fibers. Changes in tensile properties may be attributed to the highly porous nature, which provided the elongation properties, whereas the decrease in the hydrophobic quality of bare PCL decreased the tensile strength of the mats upon the incorporation of herbal extract [20]. PCL and PCL + 0.5% CA mats showed nearly the same values for all tensile properties. Meanwhile, these mats displayed significant differences in values compared to other PCL-CA fibrous mats. The physicochemical characterizations via FTIR and SEM, the mechanical properties, porosity, thermal stability, and wettability of the mats developed using dual solvent c-spinning technology showed promising results after the incorporation of the CA extract. It was observed that the developed mats could be a better choice for tissue engineering and wound healing applications.

### 3.6. Effect of Protein Denaturation

Inflammation is a complex process in wound healing. The denaturation of proteins in tissue or body fluids, whereby they lose their structure due to external factors, is considered a positive marker for inflammation. The ability of the plant extract to inhibit protein denaturation against a known protein is studied as the anti-inflammatory efficacy of the extract. In this study, as a component of anti-inflammatory properties, the ability of the CA extract inhibit heat-induced BSA denaturation was investigated. The CA extract was found to be effective in inhibiting heat-induced protein denaturation. The protein inhibition or denaturation assay was carried out for different concentrations of CA extract. A maximum inhibition of 76% was observed for 60 µg/mL, 70% for 40 µg/mL, and 64% for 20 µg/mL CA extracts, as shown in Table 4. It was observed that CA extract exerts itss anti-inflammatory properties by inhibiting the denaturation of the proteins. The study also suggested that further fractionation and purification of CA-extracted compounds can lead to more stability of proteins. Furthermore, an antibacterial study was also carried out using the disc diffusion method with four different organisms, and the results are provided in Table 4. The CA extract exhibited better antibacterial activity, which may be attributed to the presence of bioactive compounds, especially the presence of flavonoid-derived glycoside (3-methyl mannoside) present in the extract [51]. Mannose-derived glycosides are reported to possess superior antibacterial and antifungal activity against Gram-negative and Gram-positive microbial species, including *Staphylococcus aureus*, *Bacillus* spp., *Candida albicans*, and yeast, by inhibiting their lectin binding pathway.

**Figure 6:**
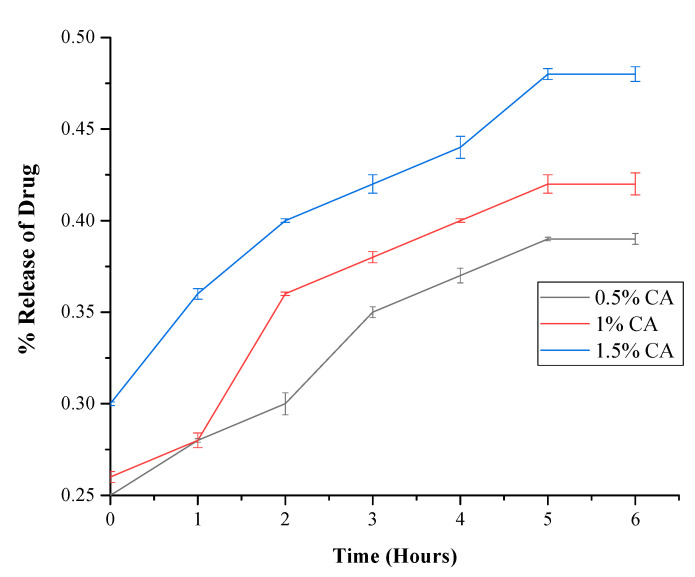
Drug release patterns of c-spun PCL-CA fibrous mats (CA: 0.5, 1, and 1.5%).

The release of CA extract from the c-spun matrices was studied in PBS solution for 6 h. Initially, CA-incorporating fibers showed a burst release within an hour. After that, all the samples exhibited a sustained release of the incorporated extract. The initial burst release of the drug may be attributed to the solubility of the extract in the medium. The mats’ more highly porous nature may tend to occupy the medium for immediate contact with the membrane enhancing the extract release. This study also revealed that the developed matrices could also be used as drug-delivery vehicles for biomedical applications. The drug release efficacies from the developed CA-incorporating mats were studied, and a sustained release of a CA extract was observed in all the samples. The drug release graph is provided in Figure 6.

### 3.7. Cell Proliferation and Adhesion

**Figure 7:**
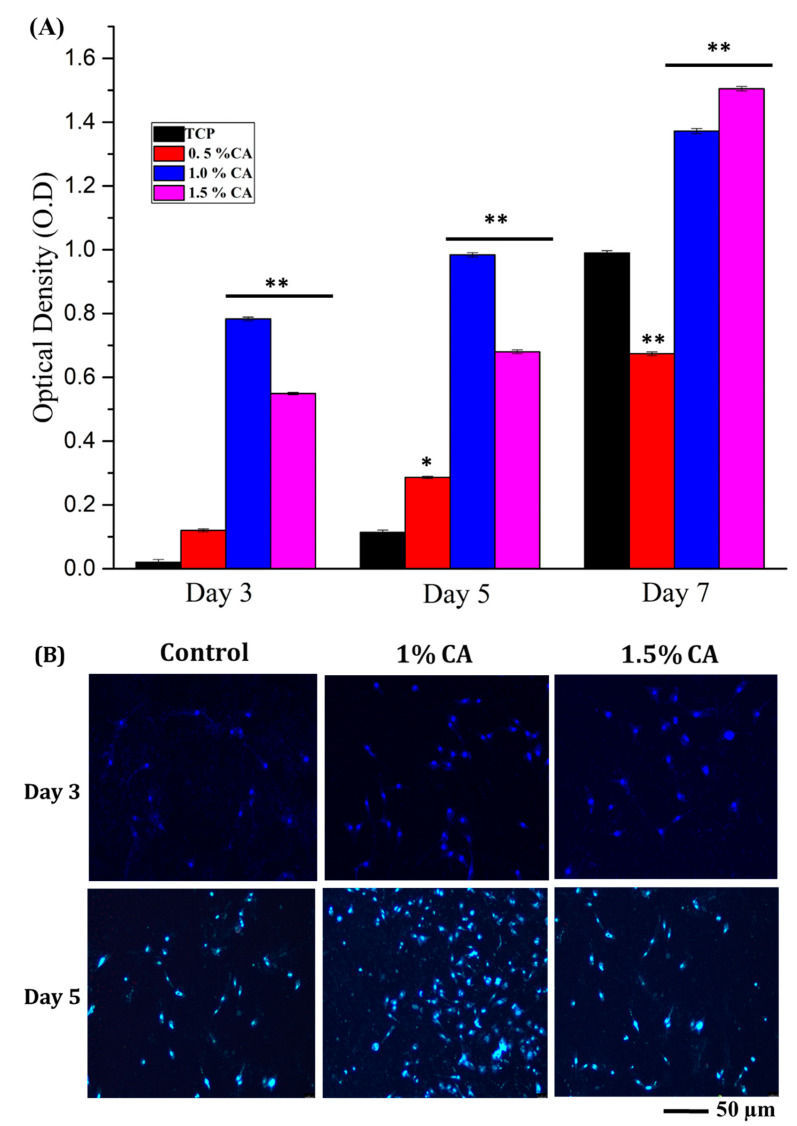
(**A**) MTT assay of NIH3T3 cell line for the developed C-spun matrices for days 3, 5, and 7. TCP—tissue culture plate; * *p* < 0.05 and ** *p* < 0.01 compared to TCP. (**B**) Cell growth and proliferation of NIH3T3 cell line on c-spun PCL and PCL-CA fibrous mats (CA: 1 and 1.5%) on days 3 and 5 (blue fluorescence from DAPI staining for nucleus).

MTT assay of the developed c-spun PCL and PCL-CA matrices was performed to study the cell proliferation efficacy of the matrices. The cell proliferation was measured as cell growth of the cells on the developed c-spun matrices at 3~7 days of culture by MTT assay (Figure 7A) and stained cell images (Figure 7B).

All the CA-incorporating mats were subjected to the proliferation assay, among which the 1% and 1.5% CA extract mats showed cell penetration and better proliferation of cells. CA extract is well known to possess many therapeutically important constituents that enhance the healing efficiency in acute wounds and have potential activity and healing diabetic wounds [23]. The presence of phenolic compounds and tannins in the CA extract can support cell growth by boosting the cellular expression factors, thereby promoting cell growth and proliferation.

**Figure 8:**
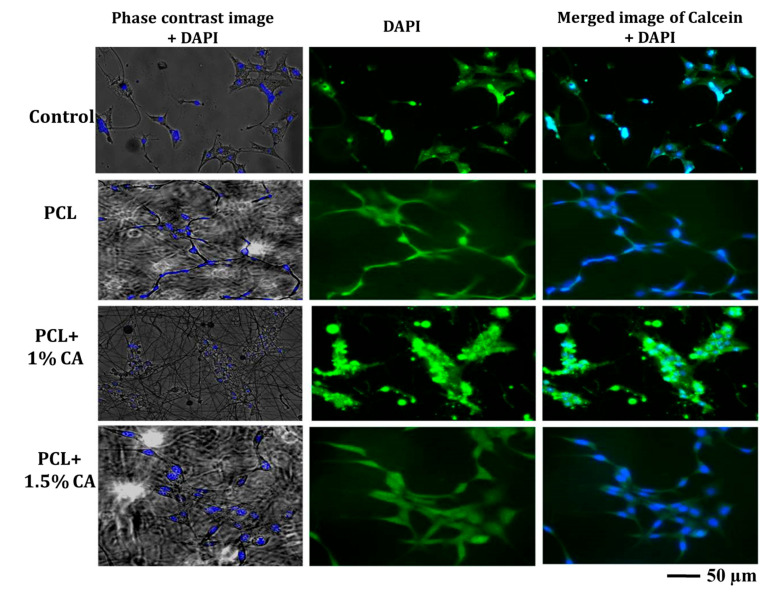
Cell morphology and adhesion of NIH3T3 cell line on c-spun PCL and PCL-CA fibrous mats (CA: 1 and 1.5%) on day 1 (control: TCP; DAPI: blue for nucleus and Calcein AM: green for cytoplasm).

DAPI and calcein AM staining showed that the cells grew and proliferated entirely on the scaffolds. This may be attributed to the surface area and porosity of the fibrous mesh, which aided better proliferation and adhesion of the cells [52,53,54]. On day 1, the cells were found to have started adhering to the scaffolds. A higher number of cells with good morphology can be seen on subsequent days of culture (Figure 8). PCL or polyester polymeric surfaces are functionalized to obtain hydrophilic property, which is commonly immobilized with amines for the effective adherence of cells. The physicochemical characterizations of the developed mats showed that they were moderately hydrophilic, which may be attributed to enhancing the cell attachment and proliferation in CA extract-incorporating mats. This showed that the 1.5% CA-incorporating PCL mat could be a good candidate for tissue engineering applications. Furthermore, we observe that the CA-PCL fiber mats do not lose their fiber morphology in physiological buffer during the cell line studies owing to their moderate hydrophilic nature and slow degrading behavior.

## 4. Conclusions

Bioactive component-incorporating nanofibrous and microfibrous materials are employed using different techniques. The present work elucidates the characteristic properties of c-spun ultrafine fibrous mat with a remarkably porous morphology achieved through dual solvent evaporation and application of heat. We developed a c-spun fibrous mat incorporating herbal extract, which can be used for tissue engineering and wound healing applications. It was observed that it was feasible to promptly develop a c-spun material with incorporating herbs. Crimping and stickiness were observed upon increasing the concentration of the extract, which may be attributed to the presence of bioactive compounds, or could also be due to the adhesion of fibers during the drying of solvents. The surface roughness and porosity were also increased, resulting in higher contact angle degrees even after the incorporation of the extract. From the SEM micrographs, it is evident that the developed mats were porous, and the incorporation of bioactive CA components resulted in a highly hydrophobic polymeric substrate to a moderately hydrophilic substrate.

Furthermore, the developed mat was subjected to cell proliferation and attachment assays, revealing that incorporating CA extracts enhanced fibroblast proliferation and better cell attachment. The c-spun matrices mimicked the extracellular matrix, supporting cellular infiltration with better attachment on the surface. The developed matrices were suitable for drug delivery and tissue engineering applications. Furthermore, the highly porous nature of the developed matrix mechanism has to be explored for application in various fields. Centrifugal spinning is one of the simplest methods for producing ultrafine fibers or nanofibers in a very short period of time. The future aspects of the present work would be tuning the parameters for producing fibers with better orientation with desired flow rate and to monitor the fiber production with pores and their strategies for desired applications in tissue engineering and other fields. Furthermore, clinical-level studies will be carried out as part of future research.

## Figures and Tables

**Table 1:** Compounds present in chloroform: methanol extract of CA by GC-MS analysis.

Peak No.	Retention Time (min)	Compound Name	Peak Area (%)	Molecular Formula	Molecular Weight
1	6.702	L- Alanyl-L- Methionine	16.64	C_8_H_16_N_2_O_3_S	291.37
2	16.641	n-Hexylmethylamine	8.56	C_7_H_17_N	115.22
3	23.755	3-methyl mannoside	58.97	C_7_H_14_O_6_	194.18
4	28.881	Di-sec-butyl-phthalate	5.03	C₁₆H₁₈O₄	278.34
5	37.086	N-Methyl-N-octadecylamine	4.67	C_19_H_41_N	283.53

**Table 2:** Porosity, thickness, and density of c-spun PCL and PCL-CA fibrous mats with different blend ratios.

Sample No	Blend Combination	Thickness	Apparent Density of the Fiber (g/cc^3^)	Porosity
1	PCL (100%)	0.425 ± 0.002	0.030 ± 0.03	97%
2	PCL + 0.5% CA	0.525 ± 0.003	0.038 ± 0.04	97%
3	PCL + 1% CA	0.425 ± 0.002	0.015 ± 0.03	98.7%
4	PCL + 1.5% CA	0.380 ± 0.004	0.040 ± 0.03	96.5%

**Table 3:** Tensile strength profiles of c-spun PCL and PCL-CA fibrous mats.

Sample No	Sample	Load (N)	Tensile Strength (MPa)	Elongation at Break (MPa)	Maximum Extension (mm)
1	PCL	2.22	1.85	63 ± 2.5	12.72
2	PCL + 0.5% CA	2.16	1.80	64 ± 2	12.87
3	PCL + 1% CA	1.43	0.96	94 ± 2.3	18.83
4	PCL + 1.5% CA	2.36	0.94	102 ± 2.3	20.40

**Table 4:** Antibacterial and anti-inflammatory effects of CA extract.

Antibacterial Activity of CA Extract on Selected Bacterial Pathogens			Effect of CA Extract on Bovine Serum Albumin (BSA) Denaturation	
Sample No	Test Organisms	CA Extract (1.5%) Zone of Inhibition (mm)	Concentration of Sample (µg/mL)	% of Inhibition
1.	*Escherichia coli*	13 mm	10	40
2.	*Bacillus cereus*	15 mm	20	64
3.	*Staphylococcus aureus*	12 mm	40	70
4.	*Pseudomonas aeruginosa*	10 mm	60	76

## Data Availability

The data presented in this study are available on request from the corresponding author.

## Supplementary Materials

The following supporting information can be downloaded at: https://www.mdpi.com/article/10.3390/polym15051293/s1, Table S1: Phytochemical Screening of CA extract; Figure S1: Gas chromatography-mass spectrometry chromatogram of chloroform methanolic extract of CA; Figure S2: Optical images of c-spun PCL and PCL-CA fibrous mats; Figure S3: Contact angle measurements of C-spun PCL and PCL-CA fiber mats. (A) PCL, (B) PCL+ 0.5% CA, (C) PCL+ 1 CA, and (D) PCL+ 1.5% CA.
