# Supplementary material for: Investigation on Centrifugally Spun Fibrous PCL/3-Methyl Mannoside Mats for Wound Healing Application
Source: Polymers (Basel). 2023 Mar 3;15(5):1293. doi: 10.3390/polym15051293 (PMC10007593; doi:10.3390/polym15051293)
Supplement: Supplementary file 1 [file polymers-15-01293-s001.zip › polymers-2210741-supplementary.pdf]

Supplementary Materials

Investigation on Centrifugally Spun Fibrous PCL/3-Methyl Mannoside Mats for Wound Healing Application

Soloman Agnes Mary [1,†], Naisini Ariram [1,†], Arun Gopinath [1], Senthil Kumar Chinnaiyan [1], Iruthayapandi Selestin Raja [2], Bindia Sahu [1], Venkateshwarapuram Rengaswami Giri Dev [3], Dong-Wook Han [2,4,*] and Balaraman Madhan [1,*]

[1] Centre for Academic and Research Excellence, CSIR-Central Leather Research Institute Adyar, Chennai 600020, India; agnufss.best@gmail.com (S.A.M.); naisini.ariram@gmail.com (N.A.); arun123gopinath@gmail.com (A.G.); csenthilmpharm@gmail.com (S.K.C.); bindiya1480@gmail.com (B.S.)
[2] BIO-IT Foundry Technology Institute, Pusan National University, Busan 46241, Republic of Korea; rajaselestin@pusan.ac.kr
[3] Department of Textile Technology, Anna University, Chennai 600025, India; vrgiridev@gmail.com
[4] Department of Cogno-Mechatronics Engineering, College of Nanoscience & Nanotechnology, Pusan National University, Busan 46241, Republic of Korea
* Correspondence: nanohan@pusan.ac.kr (D.-W.H.); madhan@clri.res.in (B.M.)
† These authors contributed equally to this work.

Table S1. Phytochemical Screening of CA extract.

S. No.	Phytoconstituents	Observation
1.	Tannins	+
2.	Flavonoids	+
3.	Terpenoids	+
4.	Saponins	-
5.	Carbohydrates	+
6.	Glycosides	+
7.	Proteins	+
8.	Anthraquinones	+
9.	Anthocyanins	+

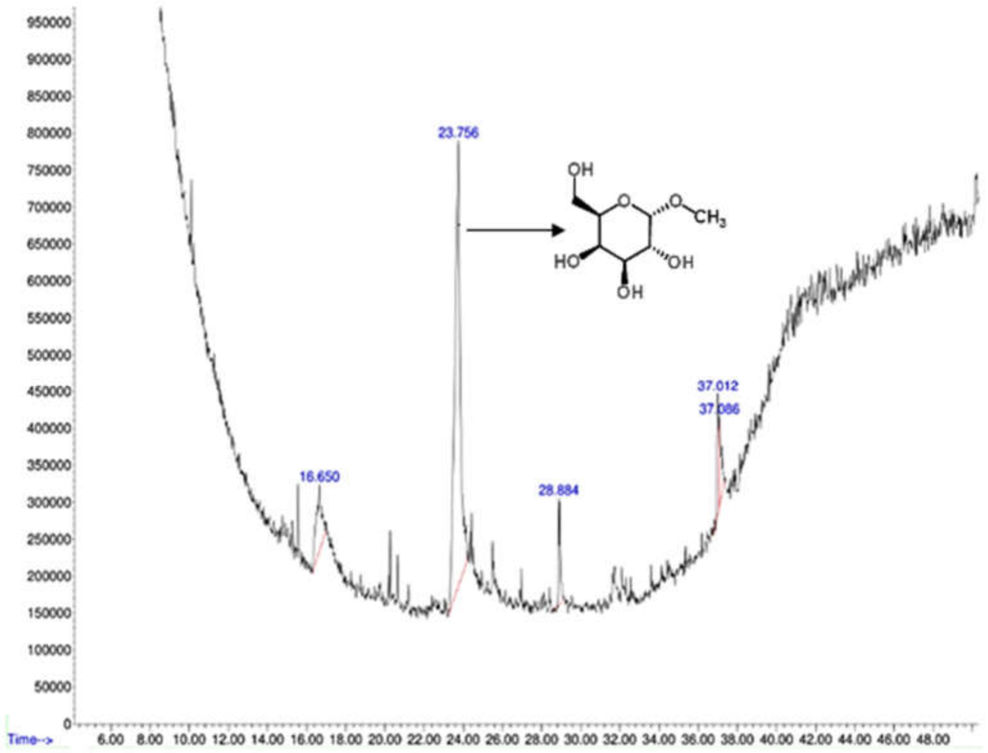

Figure S1. Gas chromatography-mass spectrometry chromatogram of chloroform methanolic extract of CA.

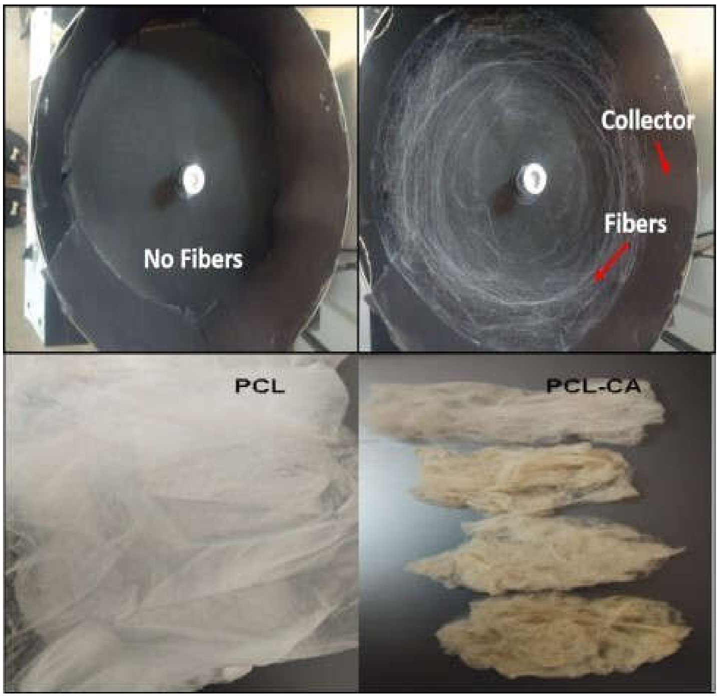

Figure S2. Optical images of c-spun PCL and PCL-CA fibrous mats.

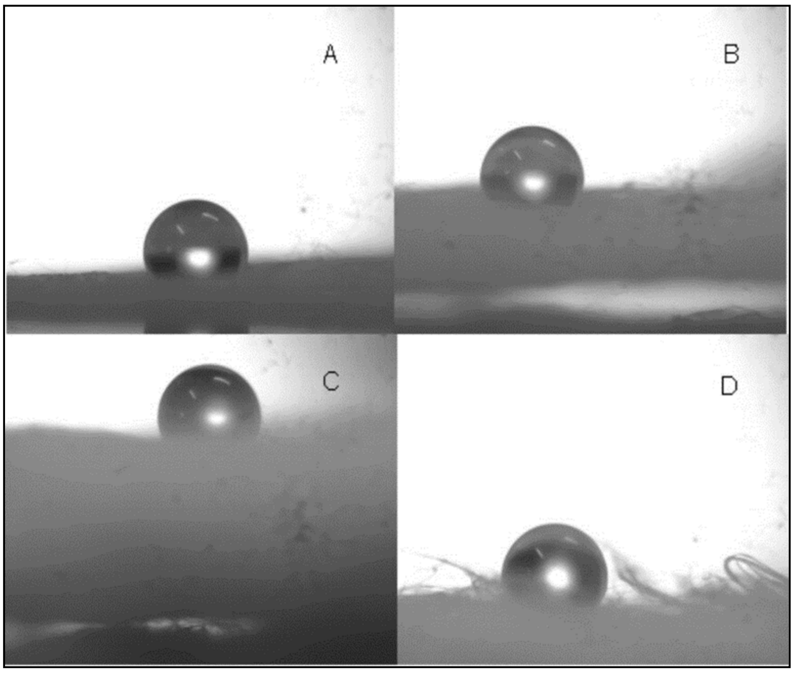

Figure S3. Contact angle measurements of C-spun PCL and PCL-CA fiber mats. (**A**) PCL, (**B**) PCL+ 0.5% CA, (**C**) PCL+ 1% CA, and (**D**) PCL+ 1.5% CA.
